# Predicting Mortality in Trauma Research: Evaluating the Performance of Trauma Scoring Tools in a South African Population

**DOI:** 10.7759/cureus.71225

**Published:** 2024-10-10

**Authors:** Christopher E Collora, Mengli Xiao, Bailey Fosdick, Hendrick J Lategan, Julia Finn, Steven G Schauer, Julia Dixon, Smitha Bhaumik, Willem Stassen, Shaheem de Vries, Craig Wylie, Nee-Kofi Mould-Millman

**Affiliations:** 1 Department of Emergency Medicine, University of Colorado School of Medicine, Aurora, USA; 2 Biostatistics and Informatics, Colorado School of Public Health, Aurora, USA; 3 Division of Surgery, Department of Surgical Sciences, Stellenbosch University, Cape Town, ZAF; 4 Department of Anesthesiology, University of Colorado School of Medicine, Aurora, USA; 5 Division of Emergency Medicine, Department of Family, Community and Emergency Care, University of Cape Town, Cape Town, ZAF; 6 Emergency Medicine, Collaborative for Emergency Care in Africa, Cape Town, ZAF; 7 Emergency Medical Services, Western Cape Government Health and Wellness, Cape Town, ZAF

**Keywords:** predicting deaths, south africa, trauma, trauma mortality prediction, trauma scoring tools, trauma severity scoring

## Abstract

Background

Trauma is a leading cause of death and disability in low-resource settings, yet trauma severity scores are seldom validated in these contexts. There is a pressing need to better characterize and compare trauma scoring tools, especially within research frameworks. This study aimed to evaluate the performance of various trauma scoring tools in predicting in-hospital mortality among trauma patients in South Africa.

Methods

This study conducted a secondary analysis of existing data from the multicenter Epidemiology and Outcomes of Prolonged Trauma Care (EpiC) study, which included 13,548 adult trauma patients aged 18 years and older, collected between August 2021 and March 2024. The predictive ability of the scoring tools was assessed by calculating the area under the receiver operating characteristic curve (AUROC) and the area under the precision-recall curve (AUPRC).

Results

The mortality rate was 2.5% (n = 298). The Kampala Trauma Score (KTS) demonstrated the highest predictive ability for seven-day in-hospital mortality, with an AUROC of 0.95 and an AUPRC of 0.53. Similarly, the Trauma and Injury Severity Score (TRISS) and the New Injury Severity Score (NISS) also exhibited strong predictive capabilities, with AUROC values of 0.96 and AUPRC values of 0.62 for TRISS and an AUROC of 0.96 and AUPRC of 0.53 for NISS. In contrast, the Revised Trauma Score and Mechanism, Glasgow Coma Scale, Age, and Arterial Pressure (MGAP) showed lower predictive performance, with AUROC values of 0.87 (AUPRC = 0.51) and 0.86 (AUPRC = 0.47), respectively.

Conclusions

The KTS exhibited optimal performance characteristics for retrospectively predicting mortality in our cohort, outperforming other scoring tools. Notably, it is also the simplest scoring tool, featuring the fewest variables compared to other trauma severity assessments. These findings highlight the necessity for external validation of trauma scoring tools in resource-limited populations to ensure their applicability and effectiveness in trauma research across diverse healthcare settings.

## Introduction

Trauma is a leading cause of death worldwide, disproportionately impacting low- and middle-income countries (LMICs) [1,2]. In South Africa, the injury mortality rate is twice the global average, with interpersonal violence and road traffic injuries responsible for the majority of trauma-related deaths [3-7]. Age-standardized mortality rates for these injury types are two and seven times higher than the global rates, respectively [3]. Trauma accounts for up to 25% of visits to public emergency facilities in South Africa, placing additional strain on resource-limited healthcare systems in the region [8].

Over the past 50 years, numerous trauma scoring tools have been developed to categorize injury severity and predict outcomes, such as post-injury mortality, among trauma patients. These tools serve multiple critical functions: some are designed for real-time trauma triage and clinical decision-making, while others focus on research and quality improvement. This report evaluates the utility of various trauma scoring tools specifically for research applications, emphasizing South Africa and other LMICs.

In research studies, trauma scoring tools quantify injury severity based on anatomical and/or physiological parameters. The Abbreviated Injury Scale (AIS), developed in 1969, assesses injury severity by anatomical structure and body region, forming the basis for the purely anatomic-based Injury Severity Score (ISS) and New ISS (NISS) [9,10]. The ISS is calculated as the sum of the squares of the highest AIS scores, while the NISS, designed to succeed the ISS, sums the squares of the three highest AIS scores, allowing for the same body region to be counted multiple times [11,12]. One limitation of these anatomic scores is that they can only be applied retrospectively once all relevant hospital and autopsy reports are available.

The Revised Trauma Score (RTS) is a well-established example of a purely physiological scoring tool [13]. Initially designed for use at the time of triage, the RTS is the weighted sum of three coded physiological variables: Glasgow Coma Scale (GCS), systolic blood pressure (SBP), and respiratory rate (RR). The Mechanism, GCS, Age, and Pressure (MGAP) score is another instrument developed for clinical trauma triage, combining mechanism and physiological variables. MGAP incorporates the mechanism of injury (MOI; blunt versus penetrating), GCS, age, and SBP.

The Kampala Trauma Score (KTS) is a simple additive hybrid score that utilizes physiological variables alongside the number of severe injuries. Specifically designed for application in resource-limited African settings, KTS calculation requires age, SBP, RR, Alert, Voice, Pain, Unresponsive (AVPU) score, and the number of serious injuries [14-16]. Finally, the Trauma and ISS (TRISS) is a hybrid scoring tool that necessitates more complex inputs, including RTS, ISS, age, and mechanism of injury. TRISS is a probability model that provides a direct mortality risk percentage rather than a point-based system for assigning trauma severity retrospectively. Notably, there are two sets of TRISS coefficients based on whether the MOI is blunt or penetrating.

The population in which trauma severity scores are applied is crucial for their accurate use and interpretation. ISS, NISS, RTS, MGAP, and TRISS were developed and initially validated in high-income countries (HICs), while KTS is among the few scoring systems derived from LMICs. Applying trauma scores designed for and validated in HICs to LMIC populations may lead to errors and misleading conclusions. For example, many HIC-derived trauma scores require advanced diagnostics and have been shown to underpredict mortality in LMIC populations [17-25]. External validation of trauma scoring tools is essential to assess their generalizability across different populations while considering significant group imbalances, with further research needed in LMIC settings [26,27].

South Africa presents a unique opportunity for external trauma score validation due to its high trauma burden and its public healthcare system, which follows a tiered referral process representative of other LMICs [28]. The objective of this study was to conduct a comprehensive comparison of six commonly utilized trauma scoring tools for predicting seven-day in-hospital mortality, specifically among South African trauma patients. The findings aim to enhance trauma research and quality improvement initiatives in South Africa and other LMICs.

## Materials and methods

Study design and data collection

This study is a retrospective secondary analysis of data from the Epidemiology and Outcomes of Prolonged Trauma Care (EpiC) study [29]. EpiC is a large, prospective, multi-site observational study conducted in the Western Cape of South Africa, aimed at examining the outcomes of resuscitation and interventions on post-traumatic injury mortality and morbidity among civilians with traumatic injuries accessing the public healthcare system. The study was carried out at six emergency care facilities, including a tertiary trauma center, two district hospitals, one regional hospital, and two community health centers. EpiC enrolls patients aged 18 and older who are admitted for moderate to severe trauma. Trained data collectors systematically abstracted data from patient charts into a Research Electronic Data Capture (REDCap) database. The collected data include injury characteristics, physiological characteristics, and the AIS for each anatomical injury. The EpiC study received approval from the Stellenbosch University Health Research Ethics Committee (project ID 14866; ref # N20/03/036), along with a secondary review by the Defense Health Agency Office of Human Research Oversight (OHRO, log numbers E01863.1a and E01863.1b).

Participant selection

EpiC patients with hospital encounters from August 29, 2021, to March 8, 2024 were included in this secondary analysis. We assessed patient demographic information (age and gender) and clinical data, including the MOI, AIS score, hospital arrival vital signs, and seven-day hospital disposition. Patients with MOI classified as burn, combined blunt and penetrating trauma, or unknown were excluded from the analysis. Additionally, patients missing data necessary for the calculation of each trauma score - such as GCS, RR, SBP, AVPU scale, or ISS - were also excluded.

Statistical analysis

Patient demographic, clinical, and trauma severity characteristics were summarized using descriptive statistics, with continuous measures presented as median and IQR, and categorical variables presented as counts and percentages. Trauma scores were calculated according to their definitions outlined in Table 1. The predictive performance of the scoring systems was evaluated based on their ability to discriminate between survivors and non-survivors, utilizing various probability prediction models, with seven-day in-hospital mortality as the primary outcome [30,31]. Additionally, the performance metrics of the trauma scores were analyzed for 30-day in-hospital mortality as a secondary outcome. This 30-day endpoint was included to facilitate comparison with data from other published studies that commonly use 30 days as the mortality endpoint, although it predominantly reflects in-hospital care rather than acute resuscitative efforts.

**Table 1:** Components of the trauma scoring systems The Age Index is defined as 0 for individuals aged 15-54 years and 1 for those aged ≥55 years. In terms of scoring interpretation, higher scores on the ISS and NISS indicate worse injury severity (↑), whereas lower scores on the MGAP, RTS, KTS, and TRISS signify worse injury severity (↓). AIS, Abbreviated Injury Scale; AVPU, Alert, Voice, Pain, Unresponsive; GCS, Glasgow Coma Scale; ISS, Injury Severity Score; KTS, Kampala Trauma Score; MGAP, Mechanism, Glasgow Coma Scale, Age, and Pressure; MOI, mechanism of injury; NISS, New Injury Severity Score; RR, respiratory rate; RTS, Revised Trauma Score; SBP, systolic blood pressure; TRISS, Trauma and Injury Severity Score

Scoring tool	Category	Input variables	Formula and definition		Score range
ISS [11]	Anatomical	AIS	AIS1^2^ + AIS2^2^ + AIS3^2^	Sum of squares for three highest AIS scores (an AIS body region can only be used once)	1-75; ↑
NISS [12]	Anatomical	AIS	AIS1^2^ + AIS2^2^ + AIS3^2^	Sum of squares for three highest AIS scores (an AIS body region can be used more than once)	1-75; ↑
MGAP [15]	Combined anatomical and physiological	MOI, GSC, Age, and SBP	MOI + GCS + Age + SBP	Simple sum of coded and non-coded demographic, physiological, and anatomical variables	3-29; ↓
RTS [13]	Physiological	SBP, RR, and GCS	0.7326 * SBP + 0.2908 * RR + 0.9368 * GCS	Weighted sum of three coded physiological variables	0-7.8408; ↓
KTS [16]	Combined anatomical and physiological	Age, SBP, RR, AVPU, and serious injuries	Age + SBP + RR + neurological status + number of serious injuries	Simple sum of coded demographic, physiological, and anatomical variables	0-10; ↓
TRISS [14]	Combined anatomical and physiological	RTS, ISS, and Age	1/(1 + e^-b^), where blunt trauma: b = 0.4499 + (0.8085) * (RTS) + (-0.0835) * (ISS) + (-1.7430) * (Age Index) and penetrating trauma: b = 2.5355 + (0.9934) * (RTS) + (-0.0651) * (ISS) + (-1.1360) * (Age Index)	Weighted sum that calculates the probability of survival based on the MOI being either blunt or penetrating	0-1; ↓

The overall ability of each scoring tool to predict mortality was assessed using the area under the receiver operating characteristic curve (AUROC). Given the significant imbalance between the number of survivors and non-survivors in our trauma cohort, we also evaluated the area under the precision-recall curve (AUPRC). The AUPRC specifically focused on predictions within the non-survivor group, which was our primary group of interest. AUPRC is generally recommended for imbalanced datasets, as it provides a more accurate assessment of a classification model’s performance [26,32-35]. Empirical AUROC and AUPRC values, along with 95% bootstrap confidence intervals, were computed. DeLong tests were used to perform all pairwise comparisons among the six AUROC values, resulting in 15 hypothesis tests [36]. Significance was assessed with an alpha level of 0.003, corresponding to a Bonferroni family-wise error rate of 0.05 across the 15 comparisons.

ROC thresholds for mortality prediction were determined based on the Youden index, targeting a sensitivity of 95% or higher. The Youden index represents the threshold value that maximizes the sum of sensitivity and specificity, giving equal weight to both. The 95% sensitivity threshold was applied to identify score cutoff values at which at least 95% of deaths were correctly identified, potentially at the expense of lower specificity. Classification performance for these mortality thresholds was measured using sensitivity, specificity, positive predictive value, negative predictive value, and overall accuracy.

Mortality probability prediction models were developed using logistic regression, natural cubic spline regression, and isotonic generalized additive regression models. The latter two nonparametric models were selected for their flexibility in modeling nonlinear relationships with mortality while ensuring that worse severity scores correspond to higher probabilities of death. In the natural cubic spline regression, we specified the model with a linear tail-restricted cubic spline function with three knots, applying penalty factors of 2, 4, and 10 to the coefficients of linear, nonlinear, and interaction terms. The three regression models were compared for each scoring system using log loss as the cost function in a five-fold cross-validation procedure.

All analyses were performed using R version 4.3.1 (R Core Team, 2023, R Foundation for Statistical Computing, Vienna, Austria) in RStudio version 2023.6.2.561 (RStudio Team, 2023, RStudio, PBC, Boston, Massachusetts, USA), utilizing the precrec, pROC, and cgam packages [37-39].

## Results

Baseline, clinical, and trauma severity characteristics of patients

**Figure 1:**
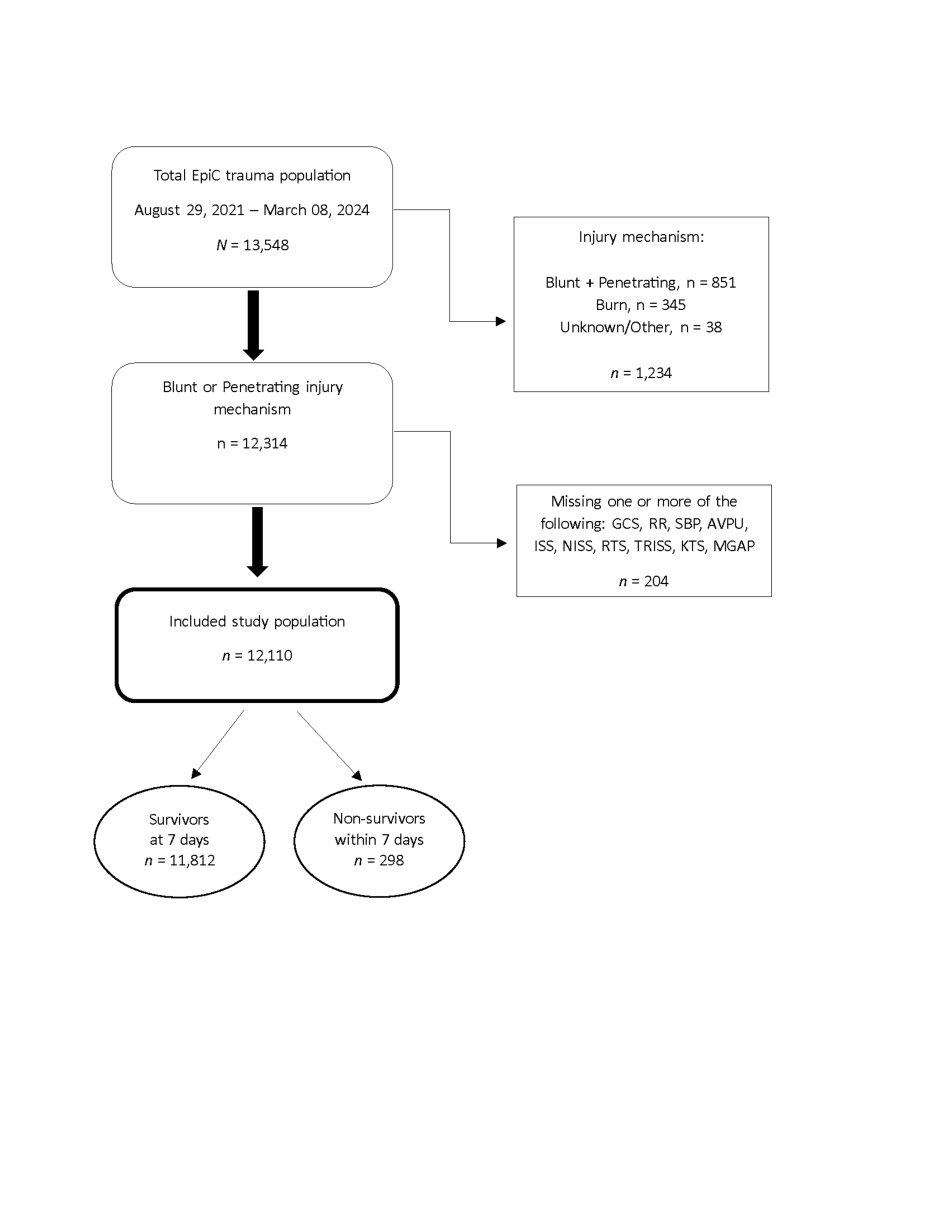
STROBE diagram outlining the patient selection criteria and the number of cases included in the study cohort AVPU, Alert, Voice, Pain, Unresponsive; GCS, Glasgow Coma Scale; ISS, Injury Severity Score; KTS, Kampala Trauma Score; MGAP, Mechanism of Injury, Glasgow Coma Scale, Age, and Arterial Pressure; NISS, New Injury Severity Score; RR, respiratory rate; RTS, Revised Trauma Score; SBP, systolic blood pressure; STROBE, Strengthening the Reporting of Observational Studies in Epidemiology; TRISS, Trauma and Injury Severity Score

Among the 13,548 EpiC patients who had a hospital encounter during the study period, 12,110 met the eligibility criteria (Figure 1). This cohort included 11,812 survivors (97.5%) and 298 non-survivors (2.5%), defined as in-hospital mortality within seven days. Patient characteristics are summarized in Table 2. The trauma patient cohort was predominantly male (80.9%, n = 9,793), with penetrating trauma identified as the leading MOI (58.8%, n = 7,119).

**Table 2:** Baseline demographic and clinical characteristics of the trauma cohort Non-survivor duration is within seven days from the time of admission. ^a^ Chi-squared tests were employed for binary and categorical variables, while Wilcoxon tests were utilized for continuous variables, all at a significance level of 0.05. ^b^ A patient may present with multiple AIS body region values, resulting in varying counts of injured body regions. The table displays the overall counts of patients with specific AIS body region values, where the denominator for the percentage (%) calculation is the total number of patients in each column, representing either the survivor or non-survivor group. AIS, Abbreviated Injury Scale; GCS, Glasgow Coma Scale; RR, respiratory rate; SBP, systolic blood pressure

Characteristic	All	Survivors	Non-survivors	p-value^a^
Population, n (%)	12,110	11,812 (97.5)	298 (2.5)	-
Sex (male)	9,793 (80.9)	9,530 (80.7)	263 (88.3)	0.001
Age median (IQR)	31 (26, 39)	31 (26, 39)	32 (27, 41)	0.046
SBP median (IQR)	124 (109, 138)	124 (110, 138)	106 (75, 138)	<0.001
RR median (IQR)	18 (16, 20)	18 (16, 20)	18 (14, 22)	0.287
GCS median (IQR)	15 (15, 15)	15 (15, 15)	6 (3, 13)	<0.001
Injury force, n (%)				<0.001
Blunt	4,992 (41.2)	4,799 (40.8)	193 (53.2)	-
Penetrating	7,118 (58.8)	6,948 (59.2)	170 (46.8)	-
Injured AIS body region, n (%)^b^				
Head/neck	4,073 (33.6)	3,863 (32.7)	210 (70.5)	<0.001
Face	3,284 (27.1)	3,204 (27.1)	80 (26.8)	0.967
Chest	3,965 (32.7)	3,814 (32.3)	151 (50.7)	<0.001
Abdomen	1,375 (11.4)	1,276 (10.8)	99 (33.2)	<0.001
Extremities	5,696 (47.0)	5,550 (47.0)	146 (49.0)	0.531
External	837 (6.9)	757 (6.4)	80 (26.8)	<0.001

In terms of injury distribution, the extremities were the most frequently injured body region (47%, n = 5,696), followed by the head and neck (33.6%, n = 4,073), chest (32.7%, n = 3,965), and face (27.1%, n = 3,284). Notably, head and neck injuries were significantly more prevalent among non-survivors (70.5%, n = 210). Furthermore, SBP and GCS scores upon arrival were significantly lower in patients who did not survive (SBP: 106 vs. 124, p < 0.001; GCS: 6 vs. 15, p < 0.001). Ultimately, a total of 298 patients died, resulting in a seven-day in-hospital mortality rate of 2.5%.

The median (IQR) scores for all six scoring tools are presented in Table 3. Scores derived from each scoring tool showed significant differences between seven-day in-hospital survivors and non-survivors (p < 0.001). The median scores for the ISS, KTS, and NISS among non-survivors indicated a classification of severe injury based on the suggested criteria. In contrast, the median scores for the MGAP and RTS in the non-survivor group were indicative of borderline moderate to severe injury.

**Table 3:** Comparison of trauma scores between survivors and non-survivors The arrow direction indicates the severity gradient, with a downward arrow (↓) signifying worse injury severity. ^a^ A Wilcoxon test was employed for continuous variables, using a significance level of 0.05. ISS, Injury Severity Score; KTS, Kampala Trauma Score; MGAP, Mechanism, Glasgow Coma Scale, Age, and Pressure; NISS, New Injury Severity Score; RTS, Revised Trauma Score; TRISS, Trauma and Injury Severity Score

Scoring systems (range)	Survivors, median (IQR)	Non-survivors, median (IQR)	p-value^a^
ISS (1-75) ↑	3.00 (1.00, 9.00)	25.00 (17.25, 30.00)	<0.001
KTS (1-10) ↓	9.00 (8.00, 9.00)	4.00 (3.00, 6.00)	<0.001
MGAP (3-29) ↓	25.00 (23.00, 27.00)	18.00 (13.00, 23.00)	<0.001
NISS (1-75) ↑	3.00 (1.00, 9.00)	43.00 (27.00, 57.00)	<0.001
RTS (0-7.84) ↓	7.84 (7.84, 7.84)	5.13 (3.36, 7.11)	<0.001
TRISS (0-1) ↓	0.99 (0.99, 1.00)	0.77 (0.29, 0.94)	<0.001

Trauma severity score patterns in patient mortality

**Figure 2:**
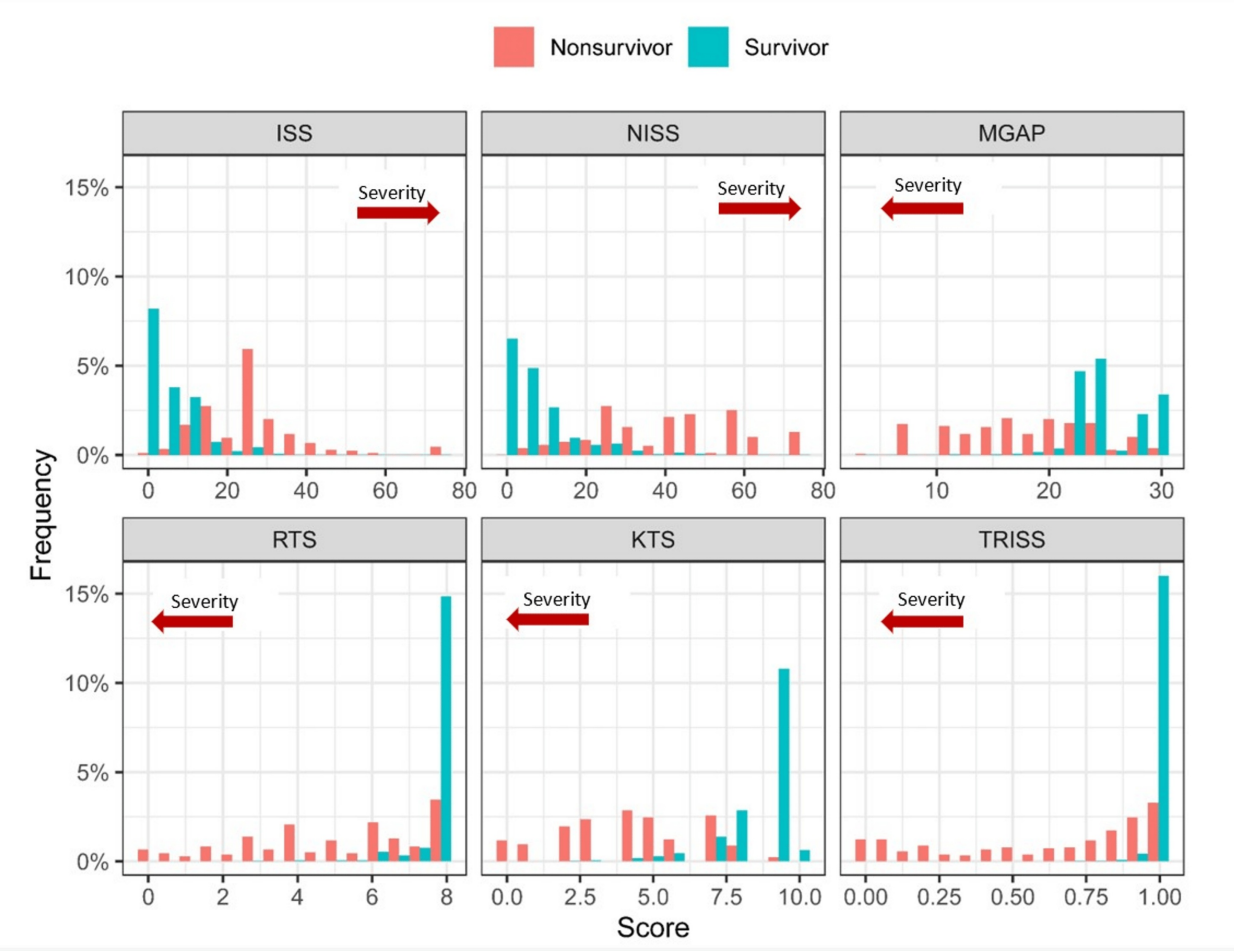
Frequency histograms illustrating the score distributions for survivor and non-survivor patient populations ISS, Injury Severity Score; KTS, Kampala Trauma Score; MGAP, Mechanism, Glasgow Coma Scale, Age, and Pressure; NISS, New Injury Severity Score; RTS, Revised Trauma Score; TRISS, Trauma and Injury Severity Score

**Figure 3:**
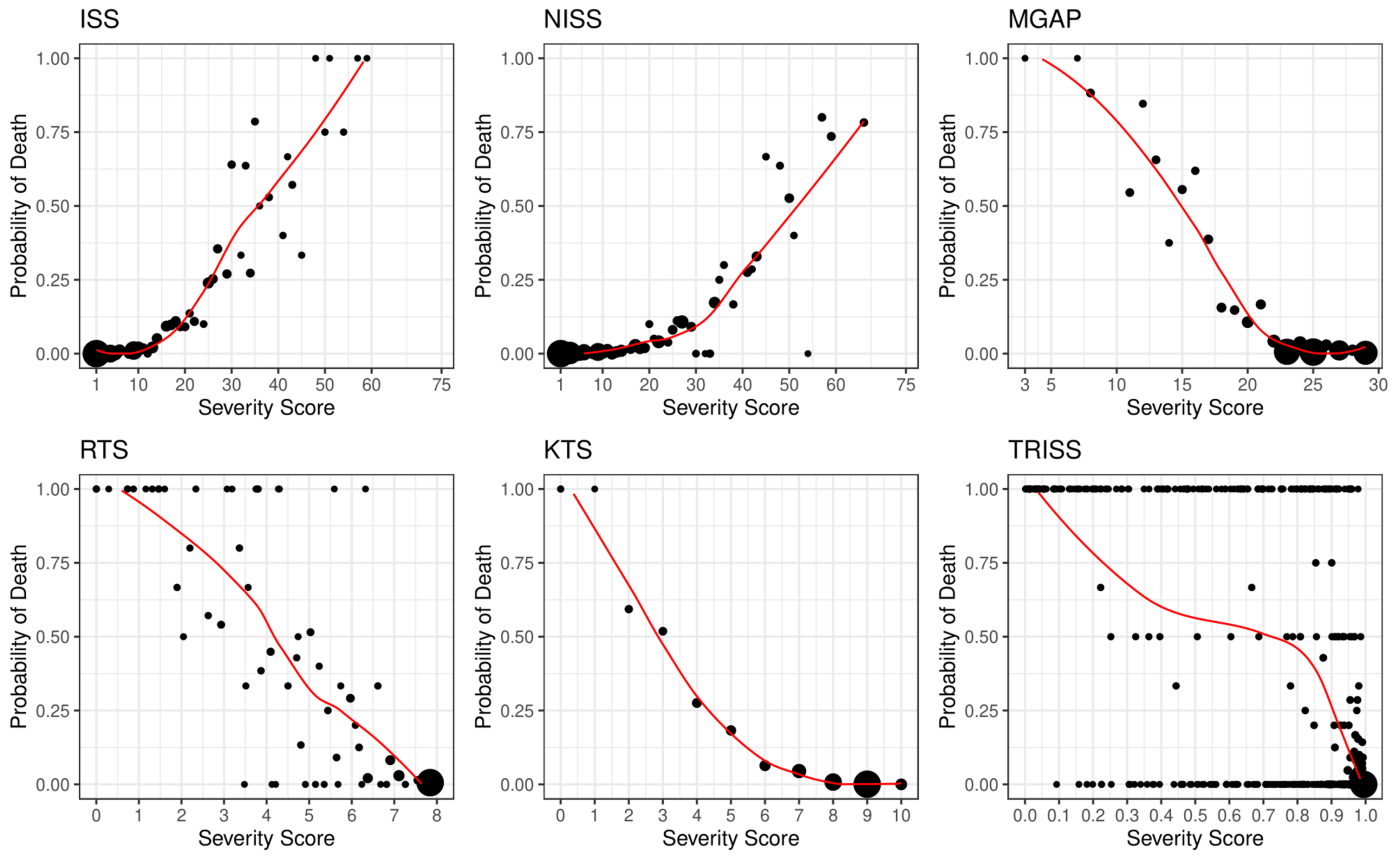
Empirical probability models predicting seven-day in-hospital mortality across various scoring systems Dots represent the empirical fraction of non-survivors, with dot size scaled according to the number of patients sharing the same score; larger dots indicate a greater number of patients. The red curves illustrate the mortality prediction trends of each empirical probability model based on locally estimated smoothing. It is important to note that, due to the limited number of patients assigned the same TRISS score, most empirical probabilities of death are either zero (indicating survival) or one (indicating death). ISS, Injury Severity Score; KTS, Kampala Trauma Score; MGAP, Mechanism, Glasgow Coma Scale, Age, and Pressure; NISS, New Injury Severity Score; RTS, Revised Trauma Score; TRISS, Trauma and Injury Severity Score

Figure 2 presents the frequency distribution of raw trauma scores categorized by survival outcome, revealing a skewness in the survivor group that suggests most patients experienced low to moderate trauma severity. In contrast, non-survivors exhibited a broader range of scores across all scoring systems. As depicted in Figure 3, the relationship between injury score and the observed probability of death was positively correlated for scoring systems where higher scores indicate greater severity (e.g., ISS and NISS) and negatively correlated for those where lower scores signify worse severity (e.g., MGAP, RTS, KTS, and TRISS). Additionally, the empirical probability of death in relation to the score was generally nonlinear across all six scoring systems.

Predictive performance of the scoring systems for in-hospital mortality

**Figure 4:**
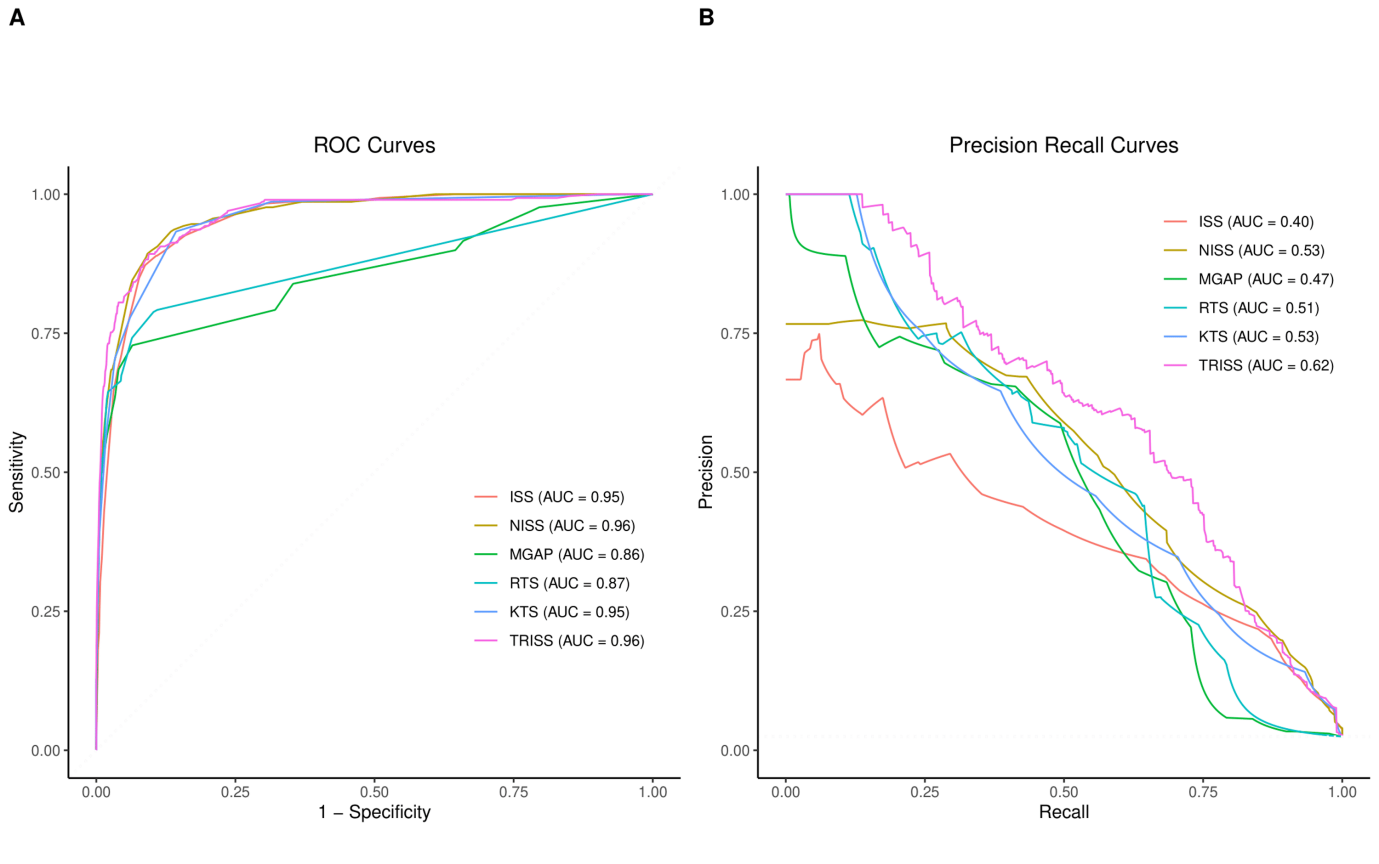
AUROC and AUPRC for all trauma scoring systems (A) ROC curves for all trauma scoring tools. Curves positioned furthest toward the upper left in the ROC space indicate higher accuracy. A greater AUC signifies improved discrimination between survivors and non-survivors. The diagonal dotted line represents the performance of a random classifier with no discriminatory ability, serving as a baseline for comparison. (B) PRCs for all trauma scoring tools. Curves that are furthest toward the upper right in the PRC space indicate higher accuracy. The horizontal dotted line represents the performance of a random classifier with no discriminatory ability, serving as a baseline for comparison. AUC, area under the curve; AUPRC, area under the precision-recall curve; AUROC, area under the receiver operating characteristic curve; ISS, Injury Severity Score; KTS, Kampala Trauma Score; MGAP, Mechanism, Glasgow Coma Scale, Age, and Pressure; NISS, New Injury Severity Score; PRC, precision-recall curve; ROC, receiver operating characteristic; RTS, Revised Trauma Score; TRISS, Trauma and Injury Severity Score

Based on the AUROC evaluation, the ISS, NISS, KTS, and TRISS exhibited the highest performance in predicting in-hospital mortality, with AUROC values of 0.95, 0.96, 0.95, and 0.96, respectively (Figure 4, Table 4). Pairwise comparisons revealed no significant differences in the AUROC among these four scoring systems, except that the AUROC for NISS was significantly higher than that for ISS (Table 5). The RTS and MGAP demonstrated significantly lower ROC performance (AUROC = 0.87 and 0.86, respectively; p < 0.001) compared to the other scoring tools. Although MGAP achieved the highest accuracy (93%) at the Youden-optimized threshold, it misclassified many actual non-survivors as survivors, as indicated by the lowest sensitivity (72.8%). In contrast, KTS, TRISS, and NISS produced the highest sensitivity based on the Youden index, with sensitivities of 93.3%, 89.3%, and 89.3%, respectively.

**Table 4:** Performance of scoring systems in predicting seven-day in-hospital mortality The “Se ≥ 95%” threshold corresponds to the cutoff that achieves at least 95% sensitivity while maximizing specificity. Se, Sp, PPV, NPV, and accuracy are reported in reference to the corresponding cutoffs. NPV results for the target 95% sensitivity cutoff are missing for the RTS scoring system, as this threshold predicts all patients to be non-survivors. AUPRC, area under the precision-recall curve; AUROC, area under the receiver operating characteristic curve; ISS, Injury Severity Score; KTS, Kampala Trauma Score; MGAP, Mechanism, Glasgow Coma Scale, Age, and Pressure; NISS, New Injury Severity Score; NPV, negative predictive value; PPV, positive predictive value; RTS, Revised Trauma Score; Se, sensitivity; Sp, specificity; TRISS, Trauma, and Injury Severity Score

Scoring system	AUROC est. (95% CI)	AUPRC est. (95% CI)	ROC threshold-specific metrics						
			Method	Cutoff	Se (%)	Sp (%)	PPV (%)	NPV (%)	Accuracy (%)
ISS	0.95 (0.94-0.96)	0.40 (0.35-0.45)	Youden	>13.5	87.2	91.2	20	99.6	91.1
			Se ≥ 95%	>8.5	97.3	72.8	8.3	99.9	73.4
NISS	0.96 (0.95-0.97)	0.53 (0.52-0.53)	Youden	>19.5	89.3	90.9	19.8	99.7	90.8
			Se ≥ 95%	>10.5	95.6	79	10.3	99.9	79.4
MGAP	0.86 (0.83-0.90)	0.47 (0.41-0.53)	Youden	<22.5	72.8	93.5	22.1	99.3	93
			Se ≥ 95%	<27.5	97	21.8	3	99.7	23.7
RTS	0.87 (0.85-0.90)	0.51 (0.46-0.57)	Youden	<7.182	78.9	89.7	16.2	99.4	89.4
			Se ≥ 95%	Inf	100	0	2.5	-	2.5
KTS	0.95 (0.94-0.96)	0.53 (0.48-0.58)	Youden	<7.5	93.3	85.7	14.1	99.8	85.9
			Se ≥ 95%	<8.5	98.7	68.5	7.3	100	69.3
TRISS	0.96 (0.954-0.97)	0.62 (0.57-0.67)	Youden	<0.98	89.3	90.6	19.3	99.7	90.6
			Se ≥ 95%	<0.99	95.3	78.6	10.1	99.8	79

**Table 5:** p-values from pairwise comparisons of AUROC values for each trauma scoring classifier using the DeLong test, which accounts for the dependencies between ROC curves Empirical AUROC values are presented in parentheses next to the scoring tool names. An alpha level of 0.003 was utilized to assess statistical significance, corresponding to a family-wise type I error rate of 0.05 across all 15 comparisons. Values in bold exceed the 0.003 threshold, indicating that the AUC values of the two scoring systems are not statistically distinct. AUROC, area under the receiver operating characteristic curve; ISS, Injury Severity Score; KTS, Kampala Trauma Score; MGAP, Mechanism, Glasgow Coma Scale, Age, and Pressure; NISS, New Injury Severity Score; RTS, Revised Trauma Score; TRISS, Trauma and Injury Severity Score.

Scoring system (AUROC)	ISS (0.95)	NISS (0.96)	MGAP (0.86)	RTS (0.87)	KTS (0.95)	TRISS (0.96)
ISS (0.95)	-	<0.001	<0.001	<0.001	0.78	0.06
NISS (0.96)	-	-	<0.001	<0.001	0.21	0.87
MGAP (0.86)	-	-	-	0.17	<0.001	<0.001
RTS (0.87)	-	-	-	-	<0.001	<0.001
KTS (0.95)	-	-	-	-	-	0.2
TRISS (0.96)	-	-	-	-	-	-

When assessing the ability to correctly predict non-survivors, the TRISS, KTS, NISS, and RTS achieved the highest precision-recall curve (PRC) values of 0.62, 0.53, 0.53, and 0.51, respectively. The PRC accounts for the significant group imbalance (a 1:40 ratio of non-survivors to survivors) by measuring the trade-off between precision (the accuracy of positive predictions) and recall (the ability to identify all positive cases). The ROC and PRC plots illustrate similar discrimination capabilities of TRISS, KTS, and NISS in comparison to the other tools (Figure 4). For 30-day mortality, a similar predictive performance of these trauma scores was observed as in our primary analysis of seven-day mortality (Table 6).

**Table 6:** Performance of scoring systems in predicting 30-day in-hospital mortality AUPRC, area under the precision-recall curve; AUROC, area under the receiver operating characteristic curve; ISS, Injury Severity Score; KTS, Kampala Trauma Score; MGAP, Mechanism, Glasgow Coma Scale, Age, and Pressure; NISS, New Injury Severity Score; NPV, negative predictive value; PPV, positive predictive value; RTS, Revised Trauma Score; Se, sensitivity; Sp, specificity; TRISS, Trauma, and Injury Severity Score

Scoring system	AUROC est. (95% CI)	AUPRC est. (95% CI)	ROC threshold-specific metrics						
			Method	Cutoff	Se (%)	Sp (%)	PPV (%)	NPV (%)	Accuracy (%)
ISS	0.95 (0.94-0.96)	0.42 (0.37-0.48)	Youden	>12.5	87.9	89.5	19.9	99.6	89.5
			Se ≥ 95%	>8.5	96.3	73.1	9.5	99.8	73.7
NISS	0.96 (0.95-0.97)	0.55 (0.55-0.55)	Youden	>19.5	89	91.2	23	99.6	91.1
			Se ≥ 95%	>10.5	95.1	79.3	11.9	99.8	79.8
MGAP	0.85 (0.82-0.87)	0.46 (0.41-0.52)	Youden	<22.5	70.3	93.7	24.8	99.1	93.1
			Se ≥ 95%	<27.5	96.5	21.9	3.5	99.5	24
RTS	0.87 (0.84-0.89)	0.51 (0.45-0.56)	Youden	<7.2	77.8	89.9	18.6	99.3	89.6
			Se ≥ 95%	Inf	100	0	2.9	-	2.9
KTS	0.95 (0.94-0.96)	0.52 (0.47-0.57)	Youden	<7.5	91.6	85.9	16.1	99.7	86.1
			Se ≥ 95%	<8.5	98	68.8	8.5	99.9	69.6
TRISS	0.96 (0.94-0.97)	0.62 (0.58-0.67)	Youden	<0.98	88.2	90.9	22.2	99.6	90.8
			Se ≥ 95%	<0.99	96	76.9	10.9	99.8	77.5

Parametric and nonparametric regression of the association between the scoring systems and in-hospital mortality

We compared various regression models to determine which method most effectively captures the relationship between trauma scores and mortality. This approach enabled us to use the best-fit model to translate a given severity score into a probability of survival. We utilized log loss to evaluate model performance, with a smaller log loss indicating a closer alignment between observed mortality and model-predicted mortality based on severity scores. The isotonic generalized additive regression model demonstrated the smallest average log loss across scoring tools, as shown in Table 7. This finding indicates that a smooth monotonic nonparametric function of severity scores provides a superior prediction of seven-day in-hospital mortality or survivorship compared to linear or linear tail-restricted nonparametric cubic spline models.

**Table 7:** Average log loss for each scoring tool and model framework in 5-fold cross-validation ISS, Injury Severity Score; KTS, Kampala Trauma Score; MGAP, Mechanism, Glasgow Coma Scale, Age, and Pressure; NISS, New Injury Severity Score; RTS, Revised Trauma Score; TRISS, Trauma and Injury Severity Score

Prediction model	ISS	NISS	MGAP	RTS	KTS	TRISS
Logistic regression	0.0701	0.0601	0.0709	0.0716	0.0621	0.0748
Natural cubic spline regression	0.0691	0.0595	0.0691	0.0579	0.0604	0.0743
Isotonic generalized additive model	0.0654	0.0582	0.0686	0.0541	0.0602	0.0732

## Discussion

We compared and validated the accuracy of the ISS, NISS, RTS, TRISS, KTS, and MGAP trauma scoring tools for predicting mortality in a South African trauma cohort. To date, no side-by-side comparisons of these six scoring tools have been performed in a single cohort of South African trauma patients, although some studies have assessed a few scores at a time [17,40-42]. Understanding the utility and limitations of these scoring systems in different contexts enables more informed application in research and potentially enhances the quality of care assessments. For instance, scores designed for rapid assessment in emergency settings, such as RTS and MGAP, may have limitations in accurately predicting mortality retrospectively. Furthermore, evaluating the out-of-sample performance of scoring systems is crucial for validating their utility in research, including reliability and generalizability across diverse healthcare settings and patient populations. Gaining insights into these contextual differences is essential for the responsible implementation and ongoing improvement of these scoring systems in trauma research [43].

Our analysis revealed that TRISS, KTS, ISS, and NISS exhibited similar high discrimination abilities, with only a 1% difference in the AUROC range, significantly outperforming RTS and MGAP. In this population, TRISS, KTS, and NISS were superior in correctly classifying non-survivors, as demonstrated by the average precision from the AUPRC. Notably, these scoring tools displayed similar performance in predicting both early (seven-day) and late (30-day) mortality, which contrasts with findings from other comparative studies [44]. Although TRISS had the highest AUPRC, KTS is particularly pragmatic for identifying severely injured patients in low-resource healthcare settings, often challenged by limited clinical data [45]. Furthermore, since KTS utilizes readily available clinical parameters that can be captured in near real-time, it highlights its potential practical utility for research screening in clinical settings, especially in clinical trials [46,47].

The TRISS calculation requires standardized evaluation and may necessitate radiographic or cross-sectional imaging due to its inclusion of the AIS-based ISS. It is not designed for mixed blunt and penetrating injury mechanisms, whereas KTS considers the presence or absence of serious injuries regardless of mechanism [24]. Given that TRISS was originally designed to categorize trauma as either blunt or penetrating, it may present challenges in assessing polytrauma cases, which comprise approximately 10% of our cohort. Overall, our findings align with previous studies that reported comparable performance between KTS and TRISS [24,45-46,48-50]. A review of KTS indicated its strong performance and generalizability across diverse patient populations, with 80% of studies reporting that KTS had equivalent or superior mortality prediction ability compared to more complex scoring tools [50].

Few prior studies assessing trauma severity outcomes have included KTS [17]. Our results contribute to the relatively limited body of literature on this scoring tool, which was uniquely developed for low-resource settings in countries like Uganda and other sub-Saharan African nations. This study supports previous findings in a larger patient population [45,46]. Notably, the AUROC for TRISS was higher than in previous South African studies and more comparable to those conducted in higher-income countries [40-42,45,48,51-53]. This suggests that TRISS can effectively serve as a retrospective predictor of mortality for blunt or penetrating injuries in an LMIC-based trauma registry, such as EpiC.

While NISS also performed well in our trauma cohort, it only considers anatomical injury severity and lacks the physiological and age components that KTS and TRISS incorporate. Not accounting for age may leave unexplained variations in mortality and potentially affect predictive ability beyond EpiC’s median age or similar cohorts. Additionally, similar to TRISS, NISS requires advanced diagnostic tools and clinical assessments to accurately identify and code all injuries. The resource demands associated with NISS and TRISS can make their applications less feasible in certain settings.

In summary, we acknowledge that relying solely on AUROC to evaluate the best mortality prediction method based on trauma scores may be inadequate. We suggest that reporting both AUROC and AUPRC offers a more comprehensive assessment of trauma scoring in predicting mortality in cohorts with significant imbalances between survivors and non-survivors. Furthermore, we have established the generalizability of trauma scoring systems in a resource-limited South African context using a large external validation dataset.

Limitations

The primary limitations of this study include its exclusive focus on seven-day mortality. However, previous research indicates that seven-day hospital mortality serves as a more appropriate endpoint for acute traumatic disease, as it better reflects mortality directly related to injury, particularly hemorrhage, compared to 30-day mortality [54,55]. Additionally, our study was confined to blunt or penetrating injuries, limiting the generalizability of our findings to other types of injury mechanisms, such as mixed injuries or burns.

Furthermore, several cases were excluded due to missing one or more variables required for calculating each trauma score; however, these exclusions were random and relatively small in number (n = 204). Lastly, we did not evaluate the relevance of these scoring tools in clinical triage or care, thereby intentionally restricting our findings and recommendations to research applications.

## Conclusions

Our findings indicate that KTS and TRISS exhibit comparable performance in predicting seven-day mortality within this South African multicenter trauma cohort. KTS is advantageous as it does not necessitate advanced diagnostic tools for injury classification and is straightforward to calculate. This study suggests the potential generalizability of both KTS and TRISS for research in other resource-limited trauma populations. Moreover, it supports the increasing evidence that KTS is at least as accurate, if not more so, in predicting mortality compared to other, more complex, and commonly utilized scoring systems.
